# Integrating machine learning and the GGE biplot for identification of climate-suitable grasspea genotypes

**DOI:** 10.3389/fpls.2025.1647903

**Published:** 2025-11-21

**Authors:** Surendra Barpete, Arpita Das, Mangla Parikh, Sonika Yumnam, Muhammad Aasim, Seyid Amjad Ali, Akanksha Singh, Ashutosh Kumar Yadav, Narayana Bhat Devate, Smita Kaul, Sudip Bhattacharya, Soumyayan Roy, Sanjeev Gupta, Shiv Kumar

**Affiliations:** 1 International Center for Agricultural Research in the Dry Areas (ICARDA)-Food Legumes Research Platform, Sehore, India; 2 Department of Genetics and Plant Breeding, Bidhan Chandra Krishi Vishwavidyalaya, Mohanpur, West Bengal, India; 3 Department of Genetics and Plant Breeding, Indira Gandhi Krishi Vishwavidyalaya, Raipur, Chhattisgarh, India; 4 Department of Genetics and Plant Breeding, Central Agricultural University, Imphal, Manipur, India; 5 Department of Precision Agriculture and Agricultural Robotics, Faculty of Agricultural Sciences and Technologies, Sivas University of Science and Technology, Sivas, Türkiye; 6 Department of Information Systems and Technologies, Bilkent University, Ankara, Türkiye; 7 International Center for Agricultural Research in the Dry Areas (ICARDA), New Delhi, India; 8 Division of Crop Sciences, Indian Council of Agricultural Research, Krishi Bhawan, New Delhi, India

**Keywords:** stability, selection index, GE interaction, machine learning, grasspea

## Abstract

Grasspea is a nutrient-rich food legume crop known for its resilience in the challenging agro-ecosystems. However, information is scanty regarding the recommendation of grasspea genotypes with respect to their suitability for both general and specific adaptations. The primary goal of the study was to delineate stable grasspea genotypes by nullifying the influence of intricate interactions among multiple traits with the environment. Additionally, the study aimed to identify suitable locations within diverse agro-climatic zones in India for future evaluation while also validating and predicting results using machine learning algorithms. From several hundred genotypes developed and tested in station trials at Amlaha, India, a panel of 64 diverse promising grasspea genotypes was identified, and their performance was subsequently assessed through multilocation testing at four diverse locations in India during 2021–2022 using the GGE biplot approach. Mean selection index of each genotype was enumerated considering multi-trait performance for better elucidation of genotype and environment ranking as well as selection of the mega-environment. The findings revealed that the environment was the primary contributor to variation across all studied traits, followed by genotype × environment interactions as the second most influential factor. Genotypes such as FLRP-B54-1-S2, Prateek, 31-GP-F3-S7, 31-GP-F3-S4, FLRP-B38-S5, 48-GP-F3-S3, and BANG-288-S2 were identified as good performers with promising multi-trait performance. Experimental results were validated using multiple performance metrics, with the Random Forest (RF) model of machine learning demonstrating superior predictive accuracy compared to the multilayer perceptron (MLP) model. Regression coefficient (*R*^2^) values ranged between 0.558 and 0.947, depending on the output variables. In conclusion, “Prateek,” “31-GP-F3-S7,” and “48-GP-F3-S3” emerged as the most stable genotypes when considering their combined yield-trait performance. These genotypes can be recommended for widespread commercial cultivation in regions where grasspea cultivation faces challenges of weather extremities.

## Introduction

Grasspea (*Lathyrus sativus* L.) is a highly adaptable and nutrient-dense cool season food legume crop, cultivated globally in resource-poor dry areas (Mahapatra et al., 2020). Its resilience to varying climates makes it a valuable component for sustainable agriculture and food security in the face of changing climatic conditions (Banerjee et al., 2022; Barpete et al., 2024). This food legume holds significant importance due to its diverse applications in human food, animal feed, and ecosystem management as an input rational crop with an ability to maintain soil health through biological nitrogen fixation. Grasspea is renowned for its high seed protein content (17.7%–49.3%), which boasts an excellent amino acid balance (19.69–23.48 g in 100 g of seed) (Rizvi et al., 2016; Sharma et al., 2022). Notably, the presence of valuable nutraceuticals, such as L-homoarginine, further enhances its nutritional value (Lambein, 2000; Das et al., 2021).

Grasspea is cultivated in rainfed dry regions of Southeast Asia, including India, as well as the Mediterranean, Middle East, and parts of Southern Europe, which are prone to weather whiplashes like the concomitant occurrence of drought and waterlogging (Barpete et al., 2023). The current total area under grasspea cultivation is approximately 0.70 million ha, with a production potential of 0.79 million tons along an average productivity of 1,130 kg per ha (Kumar et al., 2021), The plausible reason behind the decline in area under grasspea cultivation includes the stigma of neurotoxin β-N-oxalyl-L-α,β-diaminopropionic acid (β-ODAP), indeterminate growth habit, and the challenge of maintaining varietal purity due to frequent cross-pollination (Parihar et al., 2022; Tripathi et al., 2022). Identification of early maturing and stable grasspea genotypes that can harmonize optimum vegetative and reproductive growth while maximizing biomass and yield is the most seminal crop breeding property. Lack of comprehensive studies on assessing grasspea genotypes across different locations has created a pressing need to gain a deeper understanding of cultivar behavior under diverse agro-climatic conditions.

Comparing diverse genotypes in multiple environments is a crucial approach that provides vital information for the selection and recommendation of crop cultivars tailored to specific locations (Das et al., 2019; 2020). The performance of a genotype concerning quantitative traits is determined by genotypic main effect (G), environmental main effect (E), and genotype × environment interaction (GEI) (Yan et al., 2007). This GEI can lead to differential genotype ranking across environments based on the key traits’ performance, which may often mislead the selection process and further recommendation due to unfavorable traits’ association (Chatterjee et al., 2023). These challenges can be addressed through two strategies, viz., independent culling and enumeration of selection indices, both of which have been considered for the ranking and selection of genotypes (Yan and Frégeau-Reid, 2018).

Over the decade, GGE biplots have gained widespread acceptance for ensuring precise identification of ideal test environments along with successful recommendation of genotypes for general and specific areas of adaptation in numerous crops including grasspea (Sayar and Han, 2015; Chatterjee et al., 2019). Nevertheless, in earlier studies, the ranking of grasspea genotypes was focused solely on single trait performance, rather than considering the evaluation of multiple traits in accordance with the breeding objectives and specific requirements of the target environment.

Machine learning (ML)-based algorithms are increasingly recognized for their effectiveness in estimating, validating, predicting, and optimizing output variables in relation to input data (Hamdia et al., 2021; Soltis et al., 2020). Unlike traditional methods, ML models do not require strict assumptions on data distribution, sample size, or variance homogeneity, making them highly robust and flexible to complex datasets (Hair et al., 2019). Recent studies highlight ML applications in high-throughput phenotyping, yield estimation, and plant counting (Nogueira et al., 2023; Ninomiya, 2022; Barbosa et al., 2020). Additionally, deep learning and image analysis have further enhanced data interpretation, extracting valuable insights from high-dimensional sources (Najafabadi et al., 2015). Artificial intelligence (AI) is revolutionizing crop production practices by integrating weather, soil, and crop data to improve yield predictions and precision farming (Aasim et al., 2023). It also aids in understanding and preserving genetic diversity, identifying beneficial traits, and supporting efficient breeding programs (Tripodi et al., 2022; Barpete et al., 2025). Despite progress, AI/ML applications in agronomic traits remain limited (Song and Ying, 2015; Almeida et al., 2021; Khanal et al., 2024) due to the complexity of biological systems in diverse environments. However, single or hybrid AI/ML models have shown promising results in predicting plant biomass, nutrient levels, chlorophyll content, and water availability, contributing to more efficient agricultural practices (Osco et al., 2019; 2020; Teodoro et al., 2024).

However, despite the recognition of grasspea’s resilience and nutritional potential, little is known about its genotype × environment dynamics when considering multiple traits together rather than single-trait evaluations. Previous studies have not integrated the GGE biplot (Sayar and Han, 2015; Chatterjee et al., 2019) with modern ML approaches for multi-trait stability analysis in grasspea. This study aims to fill this gap by explicitly testing the hypothesis that combining the GGE biplot and ML models will improve the identification of climate-suitable genotypes and optimal testing locations in India. Therefore, the present investigation integrates three objectives: (i) to identify the stable grasspea genotypes by nullifying the effect of complex association of multiple traits; (ii) to find the best locations among the tested zones for future testing of grasspea genotypes; and (iii) to validate and predict results with the aid of decision tree-based Random Forest (RF) and neural network-based multilayer perceptron (MLP) models with six different performance metrics.

## Materials and methods

### Grasspea genotypes and multilocational testing

From the preliminary screening with 450 single plant progeny lines during the 2020–2021 season, a diverse panel of 64 promising grasspea genotypes were selected for further evaluation of the different agro-climatic zones in India. These sets of genotypes consist of advanced breeding lines and selections of Nepal, Bangladesh, and Indian origin along with two popular checks (Mahateora and Prateek) of Indian origin (**Supplementary Table S1**).

The sample size of 64 genotypes was selected to balance genetic diversity and manageability of field evaluation, ensuring sufficient statistical power (>80%) to detect GEIs based on prior variance component estimates in similar legume trials.

**Figure 1:**
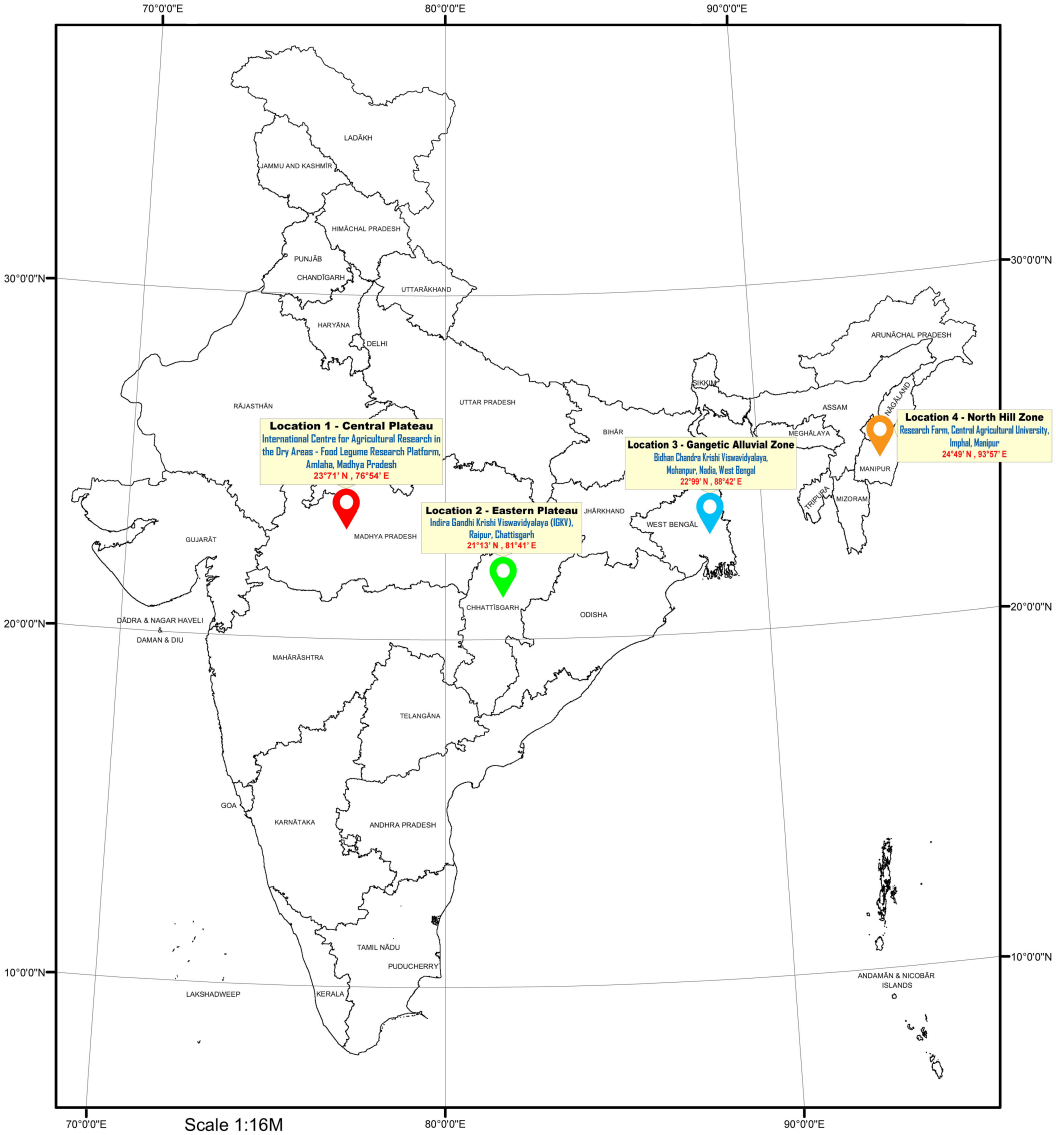
Geographical distribution of the testing locations in different agro-ecological zones in India.

**Figure 2:**
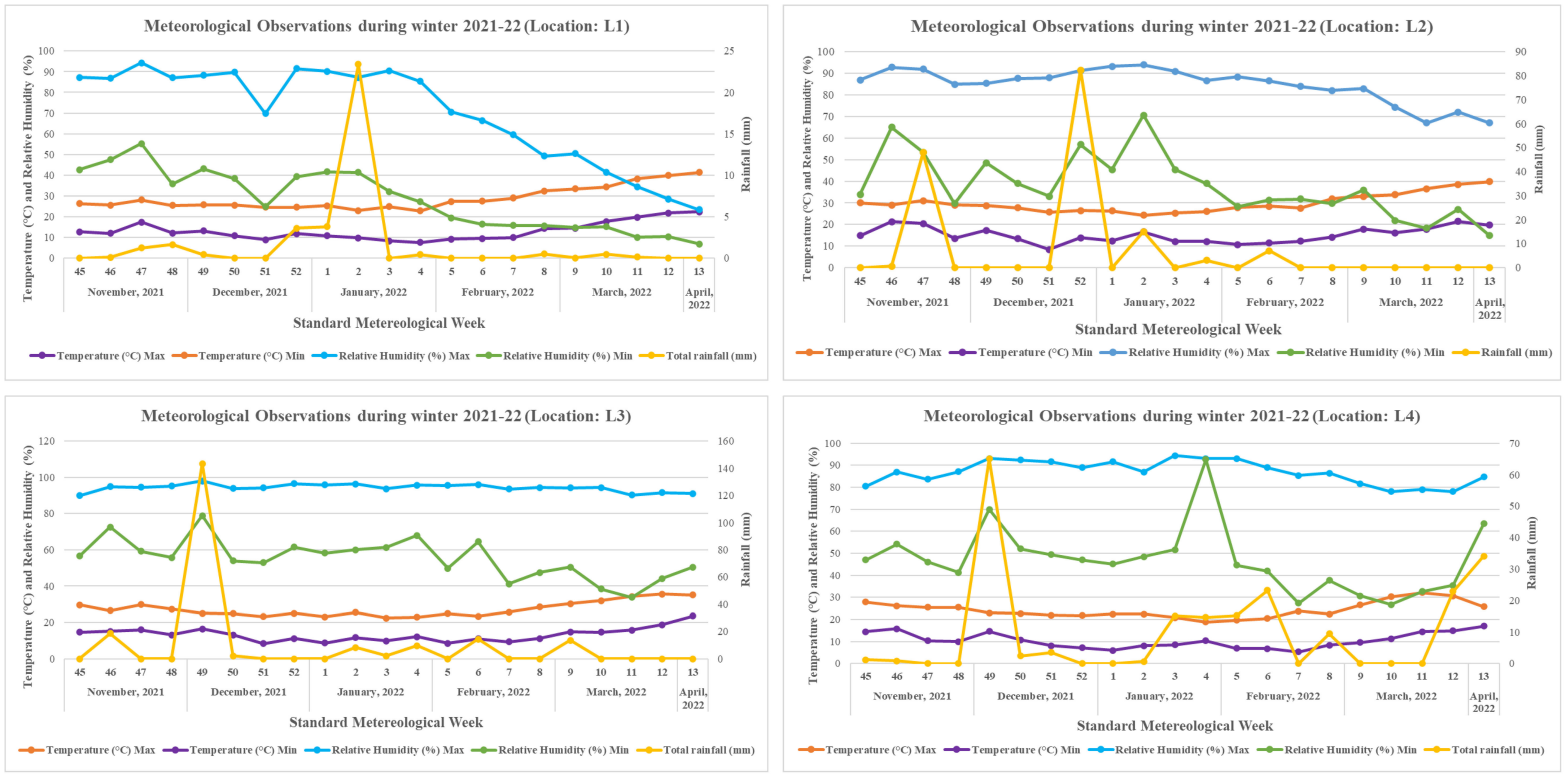
Meteorological observations during winter season of 2021–2022 across the testing locations.

Grasspea genotypes were grown over four different locations of varied agro-climatic zones in India during the winter season of 2021–2022. The testing locations represent four major grasspea-growing agro-climatic zones in India (**Figure 1**). The Central Plateau was represented by the International Center for Agricultural Research in the Dry Areas (ICARDA), Food Legume Research Platform (FLRP), Amlaha, Madhya Pradesh (henceforth L1 [latitude of 23°71′ N; longitude of 76°54′ E and 508 m above mean sea level (amsl)]) while the Indira Gandi Krishi Viswavidyalaya (IGKV), Raipur, Chhattisgarh [henceforth L2 (21°13′ N latitude; 81°41′ E longitude with 285 m amsl)] testing location falls under the Eastern Plateau region. The Gangetic Alluvial Zone was represented by a research farm under the guidance of Bidhan Chandra Krishi Viswavidyalaya, Mohanpur, West Bengal [henceforth L3 (latitude of 22°99′ N; longitude of 88°42′ E and 11 m amsl)], while the research farm of Central Agricultural University, Imphal, Manipur [henceforth L4 (latitude of 24°49′ N; longitude of 93°57′ E and 790 m amsl)] falls under the North Hill Zone of India. The weather data with respect to maximum and minimum temperature (°C) as well as % of relative humidity for each testing location during the crop season starting from sowing to harvest are depicted in **Figure 2**. At each testing location, grasspea genotypes were planted during the second fortnight of November following an Alpha lattice (8 × 8) design with two replications maintaining a proper plant geometry of 4 m row length with a 30-cm spacing between rows, having a plot size of 4.8 m^2^. Standard agronomic practices were followed across all locations to raise good crops. Data were collected and recorded using standardized protocols/procedure for days to maturity while biological and seed yield data were recorded at physiological maturity from the whole plot and were expressed in tons per ha and kg per ha, respectively, using the plot size as a factor.

### Data analysis and computation of the GGE biplot

Analysis of variance (ANOVA) was computed to reveal the effects of G, E, and GE across the testing locations. The mean significant difference within genotypes and testing locations was calculated using the LSD test at a probability level of *p* = 0.05. The relatedness of the genotypes and testing locations was represented through hierarchical clustering following the Ward method. In the present study, the Base Linear Phenotypic Selection Index (BLPSI) was enumerated considering the genetic correlation matrix of three important key traits (days to maturity, biological yield, and seed yield) necessary for varietal recommendation of grasspea genotypes. The calculated BLPSI value was plotted as a variable in the GGE biplot to aid in selection regarding the ranking and recommendation of the genotypes as per their general and specific areas of adaptation.

In the GGE biplot model, the main genotypic effect and the GE effects in different test environments were taken into consideration, while the environmental factor was nullified in the evaluation of genotypes (Yan et al., 2000). The biplot was formulated considering the first two principal components (PCs) derived from the singular value decomposition (SVD) of the mean SI. The SVD process decomposed the GGE biplot into eigenvalues of G, SI, and singular values (Yan et al., 2007).


$${\text{Y}}_{\text{ij}}=\text{μ}+{\text{e}}_{\text{j}}+\sum_{n=1}^{N}\lambda_{n}{\text{γ}}_{\text{in}}{\text{δ}}_{\text{jn}}+{\text{ϵ}}_{\text{ij}}$$

*Y_ij_* = yield of the *i*th grasspea genotypes (*i* = 1, …, *I*) in the *j*th test environments (*j* = 1, …, *J*)

*µ* = grand mean

*e_j_* = environment deviations from the grand mean

*λ_n_* = the eigen value of PC analysis axis

*γ_in_* and *δ_jn_* = genotype and environment PC scores for axis *n*

*N* = number of PCs retained in the model

*ϵ_ij_* = error term ~ *N* (0, *σ*^2^)

The current dataset was not subjected to scaling to construct an environment-centered GGE biplot (Yan and Tinker, 2006). The evaluation of genotypes was conducted using genotype-focused singular value partitioning (SVP = 1), while for the testing location evaluation, environment-focused SVP (SVP = 2) was applied (Yan, 2001). The “Average Environment Coordination” (AEC) view of the GGE biplot was created, enabling comparisons of genotypes based on mean SI integrated with stability across environments within a mega-environment (ME), following the approach introduced by Yan (2002). Concomitantly, to assess the test environments, the “discriminating power vs. representativeness” view of the GGE biplot was constructed. The ideal test environment should possess the ability to effectively discriminate among genotypes while also being representative of the ME (Yan et al., 2007). Furthermore, the “which-won-where” view of the GGE biplot was created to facilitate the detection of the superiority of genotypes across the test environments followed by grouping test environments into different MEs based on their performance (Yan and Rajcan, 2002).

### Machine learning application

In this study, decision tree-based RF models and artificial neural network-based MLP were utilized for the validation and prediction of different agronomic traits. Both models were chosen for their versatility in handling regression and classification tasks, providing robust predictive capabilities (Hesami et al., 2019; Everingham et al., 2016). RF is a decision tree model that trained multiple trees simultaneously and uses bagging, also known as bootstrap aggregation, for trained trees and showing the final outcome (Pavlov, 2019). The fundamental idea behind the RF model is presented in Equation 1.

(1) $$y=\sum_{i=1}^{n}\left(\alpha_{i}-\alpha_{i}^{*}\right)k(x,x_{i})+b$$

*y* = observed value of the data point, *n* = number of samples.

MLP is a feedforward neural network model with three completely interconnected multiple layers of processing nodes in a feedforward manner. Backpropagation is used to train the data until Equation 2 is lowered to update the error-related weights and biases (Katırcı et al., 2021).

(2) $$E=\frac{1}{K}\sum_{k=1}^{K}{\left(y_{k}-{\hat{y}}_{k}\right)}^{2}$$

*Y* = observed value of data point *k*, *k* = number of samples.

The leave-one-out cross-validation (LOO-CV) technique that is used for cross-validation was employed in this study (Webb et al., 2011). In LOO-CV, number of folds and instances of data are equal, and learning algorithm is applied to each instance individually. In this way, while using the chosen instance as the single-item test set, all other instances are set as a training set. To find the optimal hyperparameters and building the best model, a grid search approach was used. The open-source Python programming language (Van Rossum and Drake, 2009) was employed for coding with the aid of the sklearn library (Pedregosa et al., 2011). The performance of both models was evaluated by using six performance metrics Equations 3–8, providing insights into their effectiveness and suitability for different applications.

In regression-based ML analysis, regression of coefficient (*R*^2^) is the mainstay and exhibits the performance of the model by computing the proportion of variation in the dependent variable. Its value ranges from 0 to 1, and a value close to 1.0 reflects the stronger predictive accuracy of the model.

(3) $$R^{2}=1-\frac{\sum_{i=1}^{n}{\left(Y_{i}-{\hat{Y}}_{i}\right)}^{2}}{\sum_{i=1}^{n}{\left(Y_{i}-\tilde{Y}\right)}^{2}}$$

The root mean square error (RMSE) calculates the prediction errors, and low error scores demonstrate better accuracy of the model.

(4) $$MSE=\sqrt{\frac{1}{n}\sum_{i=1}^{n}{\left(Y_{i}-{\hat{Y}}_{i}\right)}^{2}}$$

The average difference between predicted and observed values is measured by the mean absolute error (MAE), and low scores present better accuracy and performance of the model.

(5) $$MAE=\frac{1}{n}\sum_{i=1}^{n}\left|Y_{i}-{\hat{Y}}_{i}\right|$$

The mean absolute percentage error (MAPE) is the prediction error given in percentage. A high MAPE score reflects a high error and shows the model’s poor performance.

(6) $$MAPE=\frac{1}{n}\sum_{i=1}^{n}|\frac{Y_{i}-{\hat{Y}}_{i}}{Y_{i}}|\times 100$$

The mean squared logarithmic error (MSLE) is the logarithmic scale of errors and is suitable for datasets with exponential relationships.

(7) $$MSLE=\frac{1}{n}\sum_{i=1}^{n}{\left(\log(Y_{i}+1)-\log({\hat{Y}}_{i}+1)\right)}^{2}$$

The median absolute error (MedAE) is based on the median of absolute differences by reducing the effect of outliers in error evaluation.

(8) $$MedAE=median(|Y_{1}-{\hat{Y}}_{1}|,\ldots ,|Y_{n}-{\hat{Y}}_{n}|)$$

*Y_i_* = measured value; ${\overset{⌢}{Y}}_{i}$ = predicted value; $\bar{Y}$ = measured value’s mean; *n* = count of samples.

## Results

### Significance of multilocational trial

ANOVA showcasing the relative contribution of each source of variation to the total variations is presented in **Table 1**. The ANOVA results indicated that the effects of genotype (G), environment (E), and their interaction (GE) were all found to be statistically significant for all the traits under investigation. Concerning the relative contribution of various components of variation, it was observed that environment has the highest contribution followed by the GE for all the traits under study. The highest contribution of environment was observed for biological yield (86.02%), whereas the contribution of GE was maximum in seed yield (14.98%). The contribution of genotype exhibited the highest effect on seed yield followed by biological yield.

**Table 1:** Analysis of variance for maturity, biological yield, and seed yield of grasspea genotypes tested across the locations.

Traits	Sources of variation	Degrees of freedom	Mean sum of square	*p*-value	% contribution
Days to maturity	Environment	3	13,223.81	<0.001	82.41
	Genotype	63	794.30	<0.001	4.95
	Environment × Genotype	189	2,028.26	<0.001	12.64
Biomass	Environment	3	814.64	<0.001	86.02
	Genotype	63	55.59	<0.001	5.87
	Environment × Genotype	189	76.80	<0.001	8.11
Seed yield	Environment	3	28,142,885.31	<0.001	78.34
	Genotype	63	2,399,725.22	<0.001	6.68
	Environment × Genotype	189	5,381,419.73	<0.001	14.98

**p < 0.01; ***p < 0.001.

### Mean performance and descriptive statistics of the grasspea genotypes over the locations

The mean performance of the tested grasspea genotypes considering their maturity, biological yield, and seed yield along with the mean BLPSI is presented in **Table 2**. Across all locations, FLRP-B54-1-S2 was the early maturity genotype (115 days) with the highest seed yield (1,740.25 kg/ha) potential. The check variety Mahateora matured early along with other six grasspea lines (BANG-147-S3, BANG-234-S1, IGC-2012-4/6-23, BANG-15-S1, IGC-2012-70/1-5, and IGC-2012-31/2-1), although all the genotypes exhibited non-significant differences with each other. Among the locations, genotypes matured early at L2 (106 days) and late in L3 (131 days). Genotypic variance (σ^2^g) varied between 2.09 (L3) and 6.24 (L4) for the days to maturity trait. Biological yield was significantly highest in genotype 23-GP-F3-S2 (10.51 t/ha) followed by IGC-2012-76/5-14 (9.94 t/ha). Genotypes exhibited the highest biological yield in L2 (11.78 t/ha), which was approximately 52% higher than the average biological yield of the genotypes over the locations. In contrast, L4 (Imphal) exhibited the lowest biological yield (6.09 t/ha), which was approximately 26% lower than the average biological yield of the tested grasspea genotypes over the locations. For this trait, genotypic variance ranged from 0.28 (L1) to 7.45 (L4).

**Table 2:** Mean performance of the grasspea genotypes across the locations.

Genotype code	Genotype/Environment	Status	Country of origin	Days to maturity	Biological yield (t/ha)	Seed yield (kg/ha)		Mean selection index (BLPSI)
Based on genotype								
G1	BANG-113-S5	Germplasm	Bangladesh	118	6.75	1,153.49	1,264.74	
G2	32-GP-F3-S2	Germplasm	Nepal	118	7.84	1,424.25	1,534.04	
G3	BANG-147-S3	Germplasm	Bangladesh	117	6.59	1,195.74	1,305.90	
G4	32-GP-F3-S5	Germplasm	Nepal	120	7.1	1,469.45	1,582.23	
G5	BANG-188-S4	Germplasm	Bangladesh	119	7.13	1,154.06	1,266.06	
G6	39-GP-F3-S2	Germplasm	Nepal	121	8.34	1,341.17	1,453.58	
G7	BANG-277-S1	Germplasm	Bangladesh	119	7.68	1,395.31	1,507.01	
G8	40-GP-F3-S3	Germplasm	Nepal	120	8.2	1,320.93	1,432.48	
G9	BANG-233-S1	Germplasm	Bangladesh	120	8.09	1,289.89	1,401.55	
G10	FLRP-B38-S5	Advanced breeding line	ICARDA	119	6.74	1,613.23	1,724.99	
G11	40-GP-F3-S6	Germplasm	Nepal	119	6.71	1,302.11	1,414.53	
G12	BANG-234-S1	Germplasm	Bangladesh	117	6.46	1,153.26	1,263.80	
G13	FLRP-B54-1-S2	Advanced breeding line	ICARDA	115	7.23	1,740.25	1,848.02	
G14	48-GP-F3-S3	Germplasm	Nepal	119	7.74	1,519.10	1,630.49	
G15	BANG-271-S2	Germplasm	Bangladesh	119	8.03	1,286.40	1,397.50	
G16	21-GP-F3-S5	Germplasm	Nepal	120	7.07	1,064.53	1,177.71	
G17	48-GP-F3-S10	Germplasm	Nepal	119	7.53	1,450.54	1,562.01	
G18	BANG-288-S2	Germplasm	Bangladesh	119	9.26	1,503.74	1,612.98	
G19	23-GP-F3-S1	Germplasm	Nepal	119	7.3	1,174.82	1,286.40	
G20	48-GP-F3-S15	Germplasm	Nepal	120	8.64	1,488.27	1,599.13	
G21	BANG-307-S2	Germplasm	Bangladesh	118	7.19	1,382.04	1,493.10	
G22	23-GP-F3-S2	Germplasm	Nepal	119	10.51	1,213.38	1,321.87	
G23	74-GP-F3-S1	Germplasm	Nepal	118	6.82	1,091.02	1,203.45	
G24	BANG-307-S3	Germplasm	Bangladesh	119	7.41	1,269.14	1,380.73	
G25	23-GP-F3-S5	Germplasm	Nepal	119	8.57	1,224.43	1,334.86	
G26	74-GP-F3-S5	Germplasm	Nepal	119	7.19	1,035.71	1,148.52	
G27	BANG-27-S2	Germplasm	Bangladesh	119	6.67	1,156.34	1,268.30	
G28	25-GP-F3-S3	Germplasm	Nepal	120	9.81	1,285.19	1,395.13	
G29	BANG-31-S6	Germplasm	Bangladesh	119	6.76	1,255.60	1,367.97	
G30	31-GP-F3-S2	Germplasm	Nepal	118	7.76	1,222.84	1,333.08	
G31	BANG-15-S1	Germplasm	Bangladesh	117	7.37	1,062.22	1,173.10	
G32	31-GP-F3-S4	Germplasm	Nepal	120	9.16	1,616.97	1,727.31	
G33	31-GP-F3-S7	Germplasm	Nepal	121	7.54	1,620.68	1,733.64	
G34	IGC-2012-70/1-8	Advanced breeding line	ICARDA	119	6.65	915.09	1,028.82	
G35	IGC-2012-31/2-37	Advanced breeding line	ICARDA	119	8.13	907.65	1,021.40	
G36	IGC-2012-6/3-36	Advanced breeding line	ICARDA	118	8.24	1,057.88	1,167.89	
G37	IGC-2012-78/4-19	Advanced breeding line	ICARDA	120	8.69	1,080.97	1,193.41	
G38	IGC-2012-76/5-42	Advanced breeding line	ICARDA	120	7.44	1,072.42	1,186.36	
G39	IGC-2012-4/6-50	Advanced breeding line	ICARDA	119	6.67	1,122.80	1,235.38	
G40	IGC-2012-2/8-8	Advanced breeding line	ICARDA	121	7.95	1,399.11	1,511.66	
G41	IGC-2012-74/10-7	Advanced breeding line	ICARDA	119	7.26	1,222.34	1,333.71	
G42	IGC-2012-88/11-50	Advanced breeding line	ICARDA	118	7.82	1,138.27	1,250.58	
G43	IGC-2012-24/12-43	Advanced breeding line	ICARDA	118	6.98	924.70	1,037.72	
G44	IGC-2012-31/2-1	Advanced breeding line	ICARDA	117	7.75	758.32	871.70	
G45	IGC-2012-6/3-42	Advanced breeding line	ICARDA	119	7.86	1,046.71	1,159.48	
G46	IGC-2012-78/4-5	Advanced breeding line	ICARDA	118	7.37	905.93	1,020.31	
G47	IGC-2012-76/5-14	Advanced breeding line	ICARDA	121	9.94	1,154.18	1,265.12	
G48	IGC-2012-4/6-8	Advanced breeding line	ICARDA	119	7.04	1,148.55	1,260.64	
G49	IGC-2012-74/10-41	Advanced breeding line	ICARDA	119	7.93	1,154.61	1,265.81	
G50	IGC-2012-24/12-26	Advanced breeding line	ICARDA	119	7.86	1,034.07	1,146.34	
G51	IGC-2012-70/1-5	Advanced breeding line	ICARDA	117	7.51	979.95	1,090.82	
G52	IGC-2012-6/3-39	Advanced breeding line	ICARDA	119	9.69	1,299.26	1,408.57	
G53	IGC-2012-31/2-44	Advanced breeding line	ICARDA	118	9.91	1,315.60	1,423.57	
G54	IGC-2012-6/3-47	Advanced breeding line	ICARDA	118	7.11	961.53	1,073.80	
G55	IGC-2012-4/6-23	Advanced breeding line	ICARDA	117	7.36	1,061.78	1,174.67	
G56	IGC-2012-14/7-44	Advanced breeding line	ICARDA	119	8.86	1,396.80	1,506.57	
G57	IGC-2012-2/8-35	Advanced breeding line	ICARDA	118	5.7	1,376.27	1,488.20	
G58	IGC-2012-73/9-5	Advanced breeding line	ICARDA	119	8.01	1,329.43	1,440.42	
G59	IGC-2012-74/10-1	Advanced breeding line	ICARDA	121	8.17	1,193.07	1,306.15	
G60	IGC-2012-6/3-43	Advanced breeding line	ICARDA	118	7.02	1,254.02	1,365.38	
G61	IGC-2012-24/12-24	Advanced breeding line	ICARDA	118	7.68	868.99	983.69	
G62	IGC-2012-67/13-25	Advanced breeding line	ICARDA	119	7.76	1,278.61	1,391.98	
G63	Mahateora	Released variety	India	117	6.65	1,440.39	1,550.37	
G64	Prateek	Released variety	India	118	7.79	1,684.00	1,794.34	
Based on locations								
L1	FLRP, Amlaha			115	6.19	2,000.71		
L2	IGKV, Raipur			106	11.78	1,003.36		
L3	BCKV, Mohanpur			131	6.81	1,112.64		
L4	CAU, Imphal			126	6.09	818.25		
LSD (5%)				6.32	0.55	283.3		
CV				5.3	14.86	13.56		
σ^2^g				0.92	0.66	16,536.58		
Gen × Loc variance				4.39	2.78	95,370.46		

**Figure 3:**
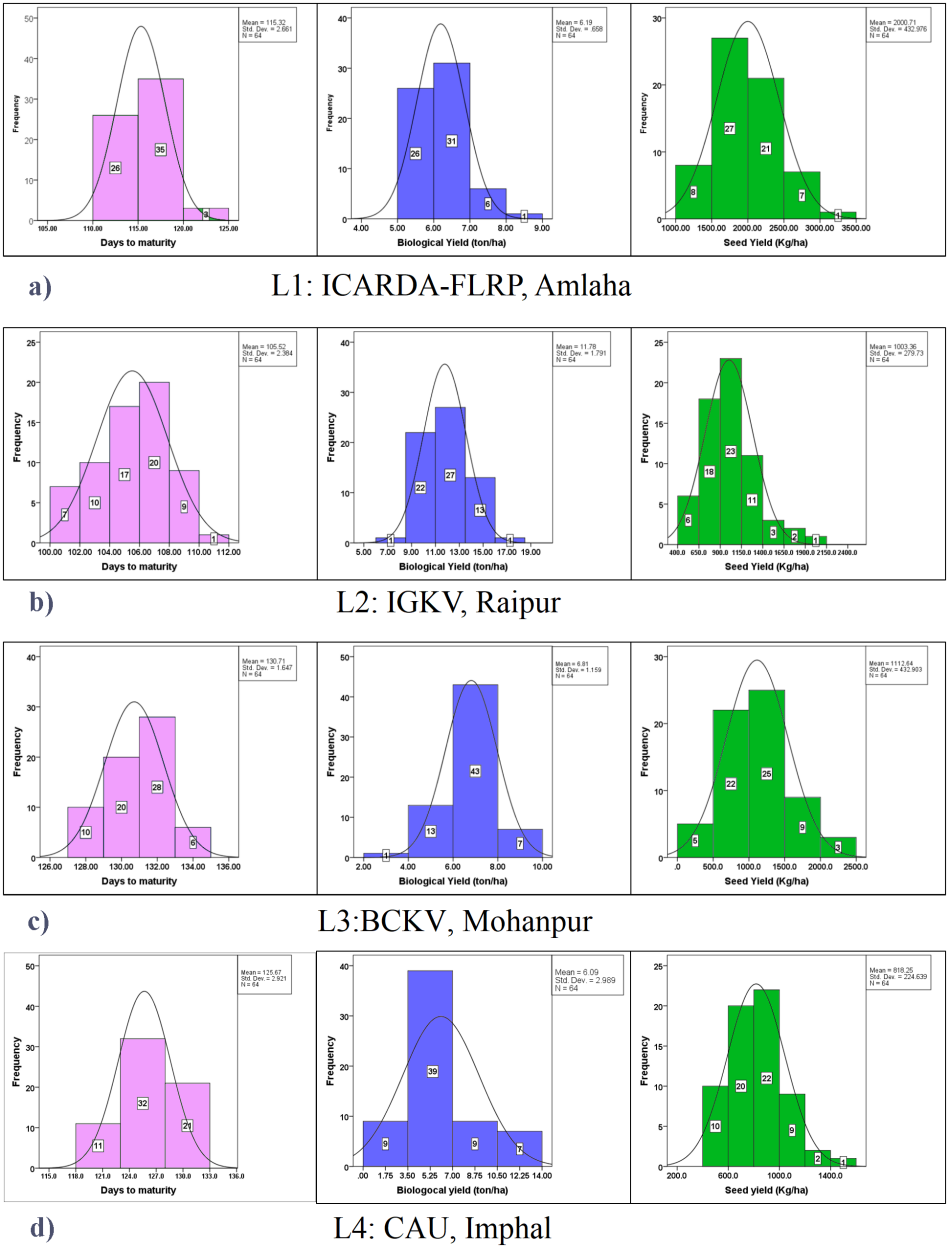
Frequency distribution of yield-attributing traits (days to maturity, biological yield: t/ha; seed yield: kg/ha) in grasspea genotypes over the locations. **(a)** Frequency distribution of yield-attributing traits in grasspea genotypes at ICARDA-FLRP, Amlaha. **(b)** Frequency distribution of yield-attributing traits in grasspea genotypes at IGKV, Raipur. **(c)** Frequency distribution of yield-attributing traits in grasspea genotypes at BCKV, Mohanpur. **(d)** Frequency distribution of yield-attributing traits in grasspea genotypes CAU, Imphal.

Among the check varieties, Prateek recorded the second highest yield (1,684 kg/ha), while maturing within 118 days over the locations. However, non-significant differences were observed between the three promising grasspea genotypes (FLRP-B54-1-S2 > Prateek > 31-GP-F3-S7) concerning yield. All these three genotypes exhibited moderate biological yield ranging from 7.23 to 7.59 t/ha. Among the locations, the highest yield was obtained in L1 (2,000.71 kg/ha), while it was the lowest in L4 (818.25 kg/ha) with a significant difference in the expression of yield potential. The highest genotypic variance as well as GEI was observed for this trait. In the present study, perusal of the data contemplating mean BLPSI reflected that FLRP-B54-1-S2 was detected with having the highest mean BLPSI (1,848.02) among all the tested grasspea genotypes, making it the top-performing genotype considering multi-trait performance. Additionally, Prateek (1,794.34), 31-GP-F3-S7 (1,733.64), 31-GP-F3-S4 (1,727.31), and FLRP-B38-S5 (1,724.99) were also ascertained as good performers considering the mean BLPSI. Among the locations, the highest mean BLPSI was observed in L1 (2,122.22) followed by L3 (1,250.16) with the lowest at L4 (950.01). In L1 (Amlaha), FLRP-B54-1-S2, Prateek, and 31-GP-F3-S7 combined early maturity with high seed yield (>1,600 kg/ha). L2 (Raipur) favored 31-GP-F3-S4, IGC-2012-31/2-44, and 23-GP-F3-S2, which recorded the highest biomass (>9 t/ha). In L3 (Mohanpur), FLRP-B38-S5 and BANG-288-S2 showed a stable seed yield performance (≥1,500 kg/ha), while in L4 (Imphal), IGC-2012-67/13-25 exhibited good adaptability with a superior yield. Overall, FLRP-B54-1-S2, Prateek, 31-GP-F3-S7, and 48-GP-F3-S3 consistently ranked highest across most environments, with seed yield advantages of 8%–15% over the trial mean (**Table 2**; **Figure 3**). In the present dataset, it was observed that in L1, out of 64 genotypes, 26 genotypes matured within 105–106 days while 3 genotypes matured beyond 120 days (**Figure 3**). Interestingly, in L2, maximum genotypic classes were observed and seven genotypes matured within 102 days. In L3, the genotypes matured within the range of 127–133 days while 10 genotypes matured early (127–128 days). In L4, only three classes were obtained, where only 11 genotypes matured within 121 days. In the case of biological yield, in L2, genotypes were divided into five classes, while four classes were observed in the rest of the locations. In contrast, in the case of seed yield, the highest classes were observed in L2 (seven classes) followed by six classes in L4. All the traits exhibited normal distribution over the locations.

**Figure 4:**
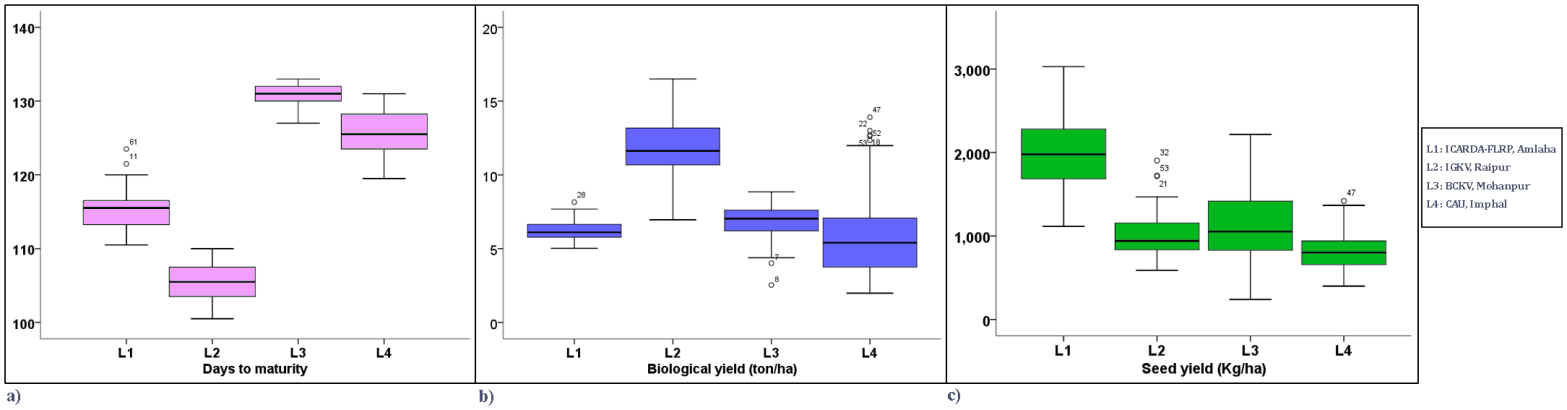
Boxplot view of yield-attributing traits of the grasspea genotypes over the locations. **(a)** Boxplot view of days to maturity of the grasspea genotypes over the locations. **(b)** Boxplot view of biological yield (t/ha) of the grasspea genotypes over the locations. **(c)** Boxplot view of seed yield (kg/ha) of the grasspea genotypes over the locations.

Boxplot analysis represented the distribution of environments concerning three yield components over the locations (**Figure 4**). For days to maturity, consistent performances were seen in L2. For biological yield and seed yield, a congruous performance was observed in L4 and L1, respectively. In the case of biological yield, L4 and L1 exhibited a stable performance with relatively low variation. Similarly, for seed yield, L4 demonstrated the most uniform performance, suggesting greater consistency in this trait.

Genetic parameters of the 64 grasspea genotypes for three traits over the locations are presented in **Table 3**. The mean value for days to maturity was found to be 119 days, and across all the genotypes and locations, the maturity period ranged from 115 to 122 days. In the case of biological trait, moderate variability was observed. Maximum variability was reflected in the case of seed yield and varied between 758 and 1,740 kg/ha. Heritability for maturity (76%) and biological yield (67%) was high, whereas for seed yield, it was moderate (58%).

**Table 3:** Descriptive statistics of the tested grasspea genotypes over the locations.

Traits	Mean	Range		GCV	PCV	Heritability	Genetic advance (GA)	GA as % over mean
		Min	Max					
Days to maturity	119	115	122	0.8	0.92	0.76	1.72	1.44
Biological yield (kg/ha)	7.72	5.7	10.51	10.53	12.87	0.67	1.37	17.76
Seed yield (kg/ha)	1,233.74	758.32	1,740.25	10.42	13.69	0.58	201.72	16.35

### Genotypic appraisal over the location considering mean vs. stability

**Figure 5:**
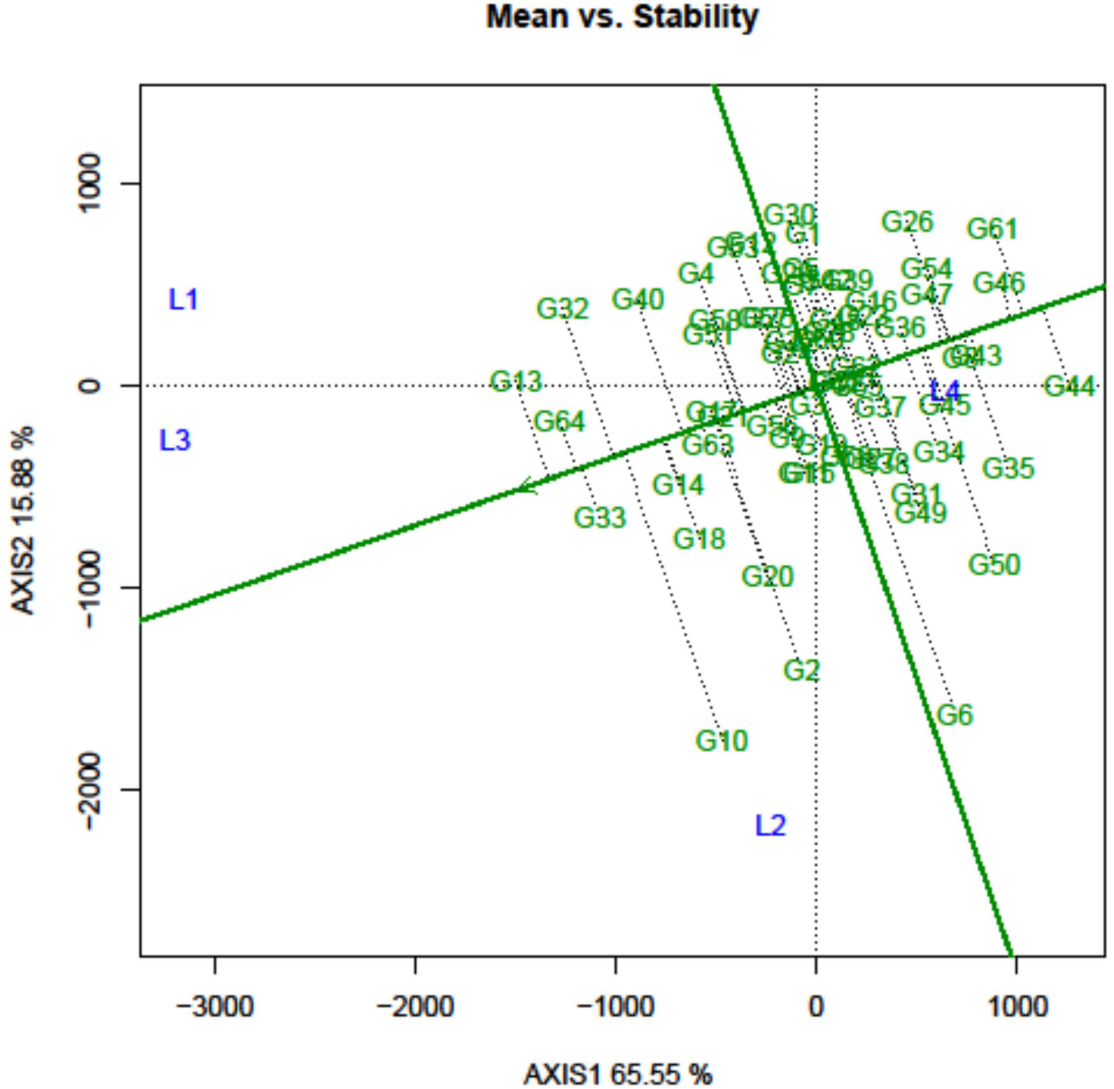
Mean performance and stability of the grasspea genotypes tested across different locations.

The mean performance and stability of the grasspea genotype across different locations considering their mean SI were visually represented using the AEC view of the GGE biplot (**Figure 5**). In essence, the AEC coordination view of the GGE biplot is a genotype-metric-preserving biplot with an SVP equal to 1. It allows for the visualization of genotype discrimination considering their mean performance. In this graph, the first two PCs enabled to explain 80.93% of the total variation considering mean SI.

**Figure 6:**
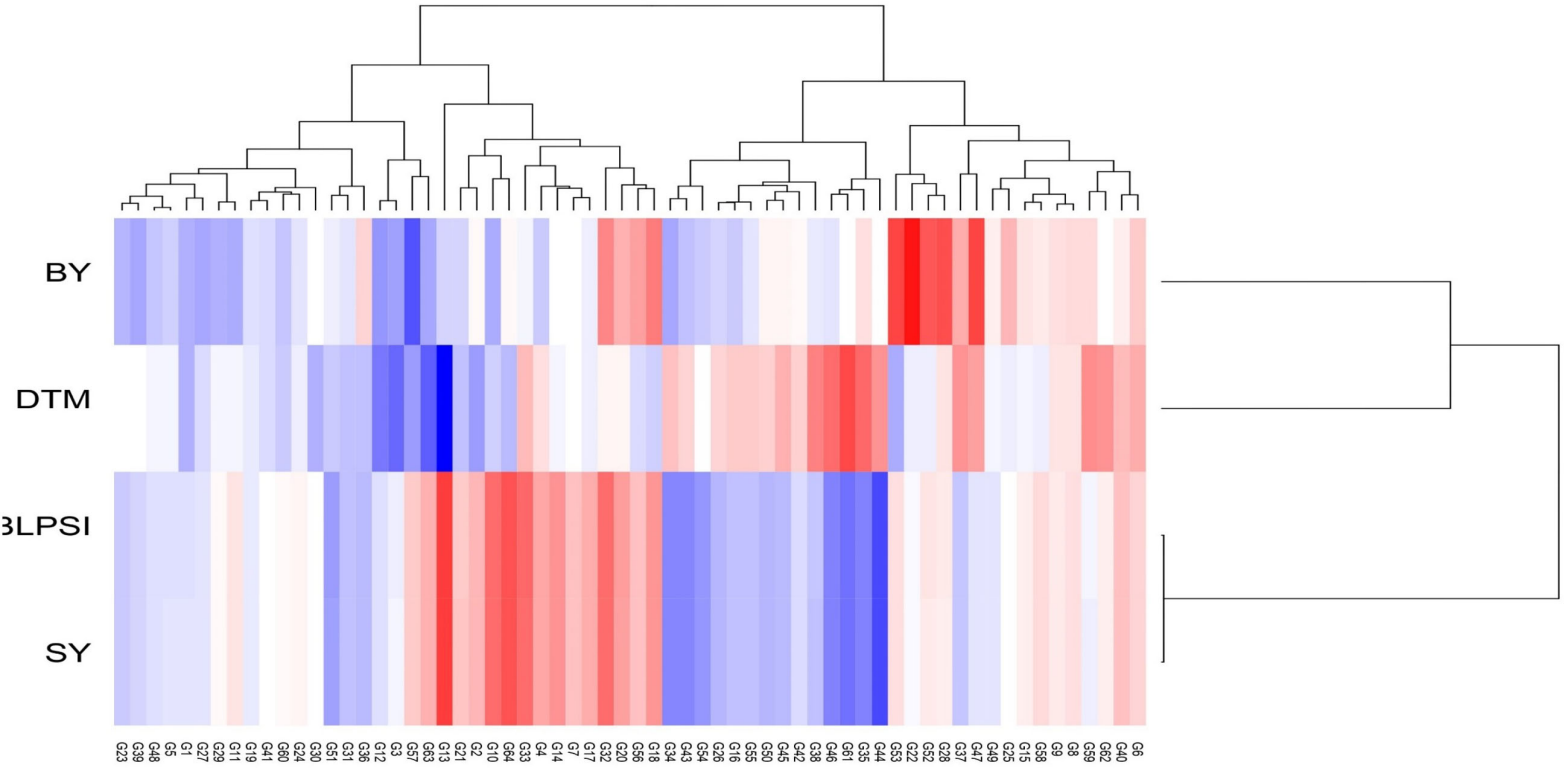
Hierarchical clustering of grasspea genotypes based on days to maturity (DTM), biological yield (BY), seed yield (SY), and the BLPSI over the tested locations.

The graph’s single arrowhead line represents the AEC abscissa, which passes through the biplot origin and indicates a higher mean SI value of the grasspea genotypes, expressing their overall superiority. The perpendicular line to the AEC, extending outward from the biplot origin, is referred to as the “AEC ordinates”, serving as an indicator of the stability of the genotypes. Inversely, genotypes with longer vector lengths from the AEC abscissa exhibited lower stability, while those with shorter vector lengths were considered more stable. Genotypes FLRP-B54-1-S2 (G13), Prateek (G64), 31-GP-F3-S7 (G33), 31-GP-F3-S4 (G32), FLRP-B38-S5 (G10), 48-GP-F3-S3 (G14), and BANG-288-S2 (G18) exhibited strong multi-trait performance, as they were positioned favorably concerning the “AEC ordinate.” On the other hand, genotypes IGC-2012-31/2-1 (G44), IGC-2012-24/12-24 (G61), and IGC-2012-78/4-5 (G46) performed poorly, as they were positioned in the opposite direction to the “AEC ordinate.” Within the group of good performers, Prateek (G64) and 31-GP-F3-S7 (G33) stood out as the most stable genotypes, as they had shorter projections from the “AEC abscissa.” Despite being the best performer, FLRP-B54-1-S2 (G13) was characterized as an unstable genotype. The present study revealed that Prateek (G64) and 31-GP-F3-S7 (G33) emerged as the most ideal genotypes due to their excellent performance in terms of combined yield-duration profile as well as good stability. The genotypes positioned close to the ideal genotype were regarded as desirable genotypes, and the distance between them is measured using the Euclidean distance. Consequently, 48-GP-F3-S3 (G14) was identified as a desirable genotype because of its proximity to the ideal genotype and nearly stable response in terms of multi-trait performance. Using the mean SI, the tested grasspea genotypes were categorized into seven clusters with promising grasspea genotypes (G13), (G64), (G33), (G32), (G10), (G14), and (G18) in cluster I (**Figure 6**).

### Environment evaluation following discriminativeness vs. representativeness

**Figure 7:**
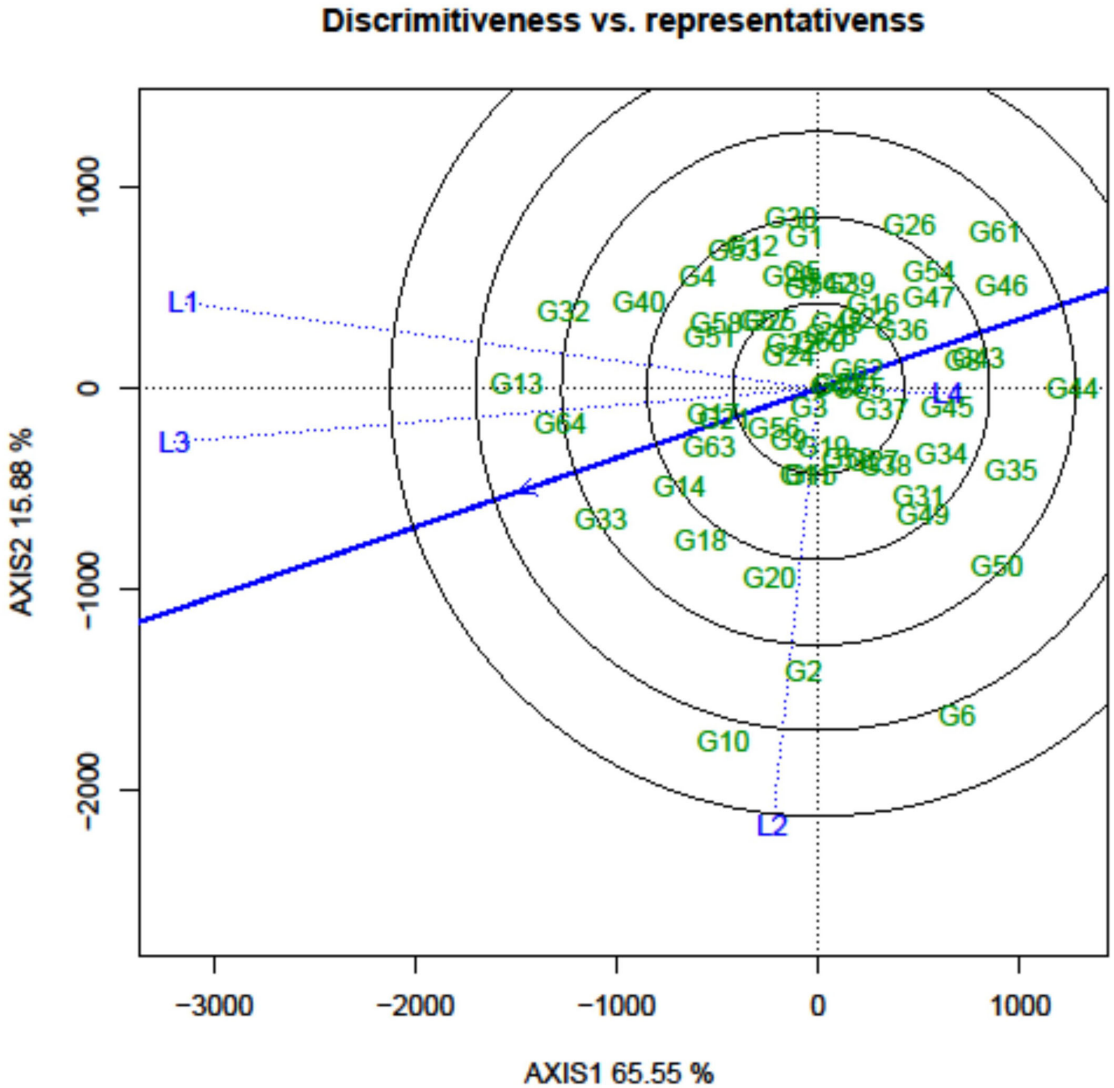
GGE biplot analysis: discriminativeness vs. representativeness of grasspea genotypes tested across different locations.

In the GGE biplot approach, the critical factors for identifying desirable testing locations and eliminating redundant ones are discrimination power (ability to discriminate genotypes), representativeness (ability to represent corresponding MEs), and the desirability index. In the “discriminativeness vs. representativeness” view of the GGE biplot, the lines connecting the test environments are referred to as environment vectors. From the graph, it was observed that L1 (Amlaha) and L3 (Mohanpur) revealed an acute angle with each other, while the two remaining locations indicated an obtuse angle (**Figure 7**).

The information rendered by L4 (Imphal) was distinct from the other locations. Thus, it can be deduced that L1 and L3 environments were positively correlated and had closer relationships with each other. The presence of a close association between these environments suggested that similar information could be obtained regarding genotype performance from these environments.In contrast, an obtuse angle was observed between L1 and L3 on one side and L2 and L4 on the other, indicating a negative association and distant relationships among these environments. The length of the environmental vectors generally represents the discriminating power of the test environments. Therefore, among the four testing locations, L3 (Mohanpur) followed by L1 (Amlaha) were the most discriminative whereas L4 (Imphal) was the least discriminative environment (**Table 4**).

**Table 4:** Evaluation parameter of the testing environment.

Location	Discriminating power	Representativeness	Desirability index
L1	7.29	0.79	6.76
L2	7.07	0.47	5.81
L3	7.32	0.87	7.22
L4	4.17	0.91	3.41

The least discriminative location is also considered as the least informative; thus, this environment can be considered as a redundant testing location for the assessment of grasspea genotypes. The indication of environmental representativeness is typically conveyed by the angle existing between the environment vectors and the AEC. It was observed that L4 (Imphal) reflected with the smallest angle between AEC and was thus identified as the most representative testing location. The desirability index of the test locations encompasses the combined outcome of both discriminative capability and representativeness. L3 (Mohanpur) with the highest desirability index was identified as the ideal testing location for the testing of mini core collection or advance breeding materials as well as for selecting genotypes with general adaptability. However, locations with good discrimination power and less representativeness such as L2 (Raipur) would be meaningful for winnowing genotypes with a specific adaptation. The relatedness of the genotypes and testing locations was represented through hierarchical clustering based on days to maturity, biological yield, and seed yield of the tested grasspea genotypes. All the testing locations were grouped into two clusters (**Figure 6**).

### Delineation of the mega-environment and the winning genotypes

**Figure 8:**
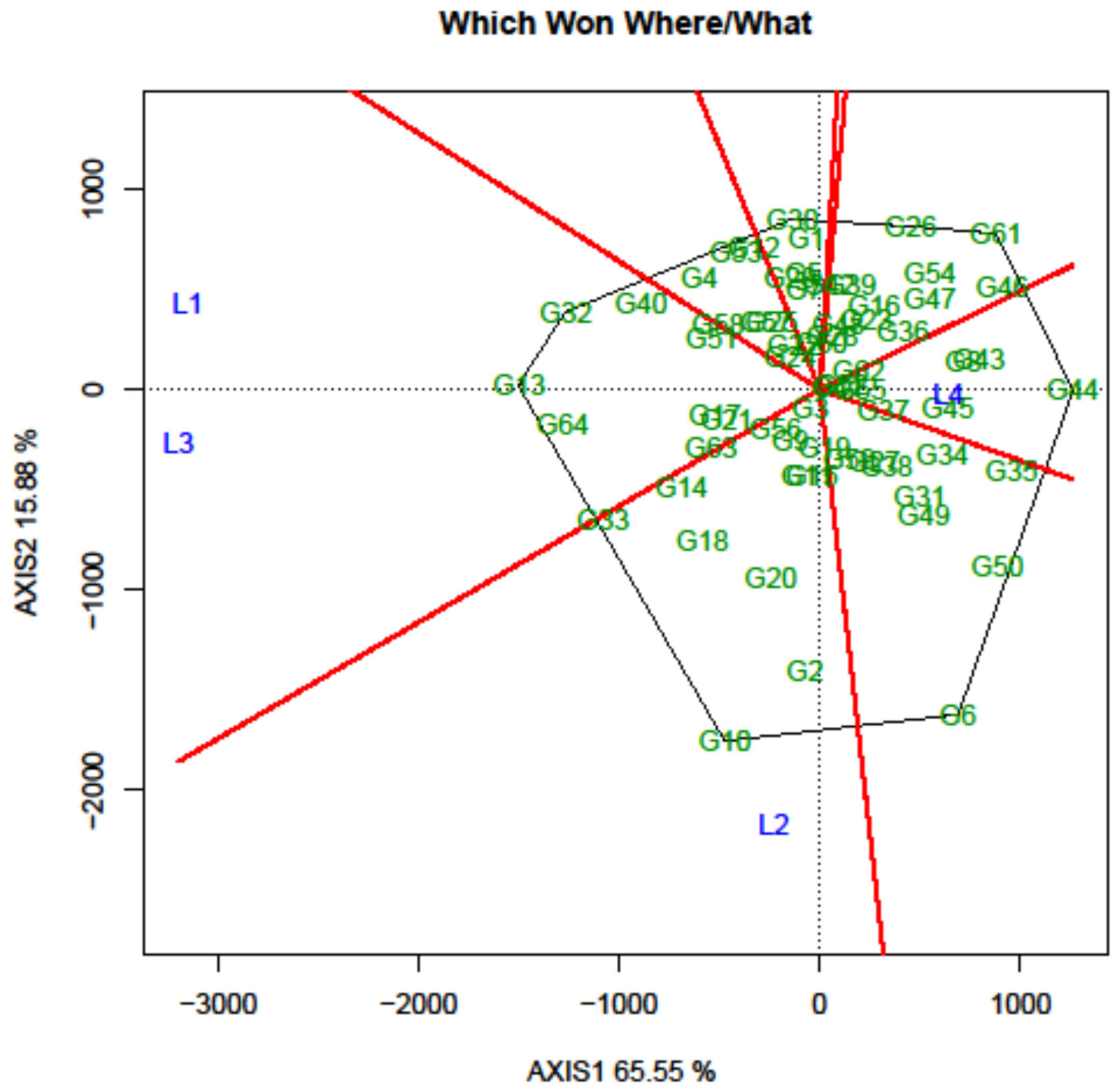
Which-won-where pattern of grasspea genotypes in the GGE biplot analysis tested over the locations.

Another crucial aspect of the GGE biplot involves the identification of genotypes suitable for test environments through the graphical depiction known as the “which-won-where” representation. Initially, a polygon was sketched around the genotypes located farthest from the biplot origin, encompassing all the remaining genotypes within its boundaries. Genotypes situated at the polygon’s vertices represent either the most superior or the least favorable performers in one or multiple environments. In the current study, the biplot proved highly informative in effectively distinguishing between environments and displaying a well-distributed polygon (**Figure 8**). The equality lines divided the graph into seven sectors, with all 4 locations being situated within three of these sectors, which could be designated as “MEs”. The first ME constituted of L1 (Amlaha) and L3 (Mohanpur), and the second and third ME consisted of single location L2 (Raipur) and L4 (Imphal), respectively. The analysis revealed that FLRP-B54-1-S2 (G13) emerged as the winning genotype in ME-I, while in ME-II, FLRP-B38-S5 (G10) was detected as the winning genotype. In the case of ME-III, the genotype positioned in the outer vertex was the poor-performing one; therefore, IGC-2012-67/13-25 (G62) present on the vertex towards the center was considered as the winning genotype.

### Machine learning modeling and delimitation of suitable genotypes of grasspea

**Figure 9:**
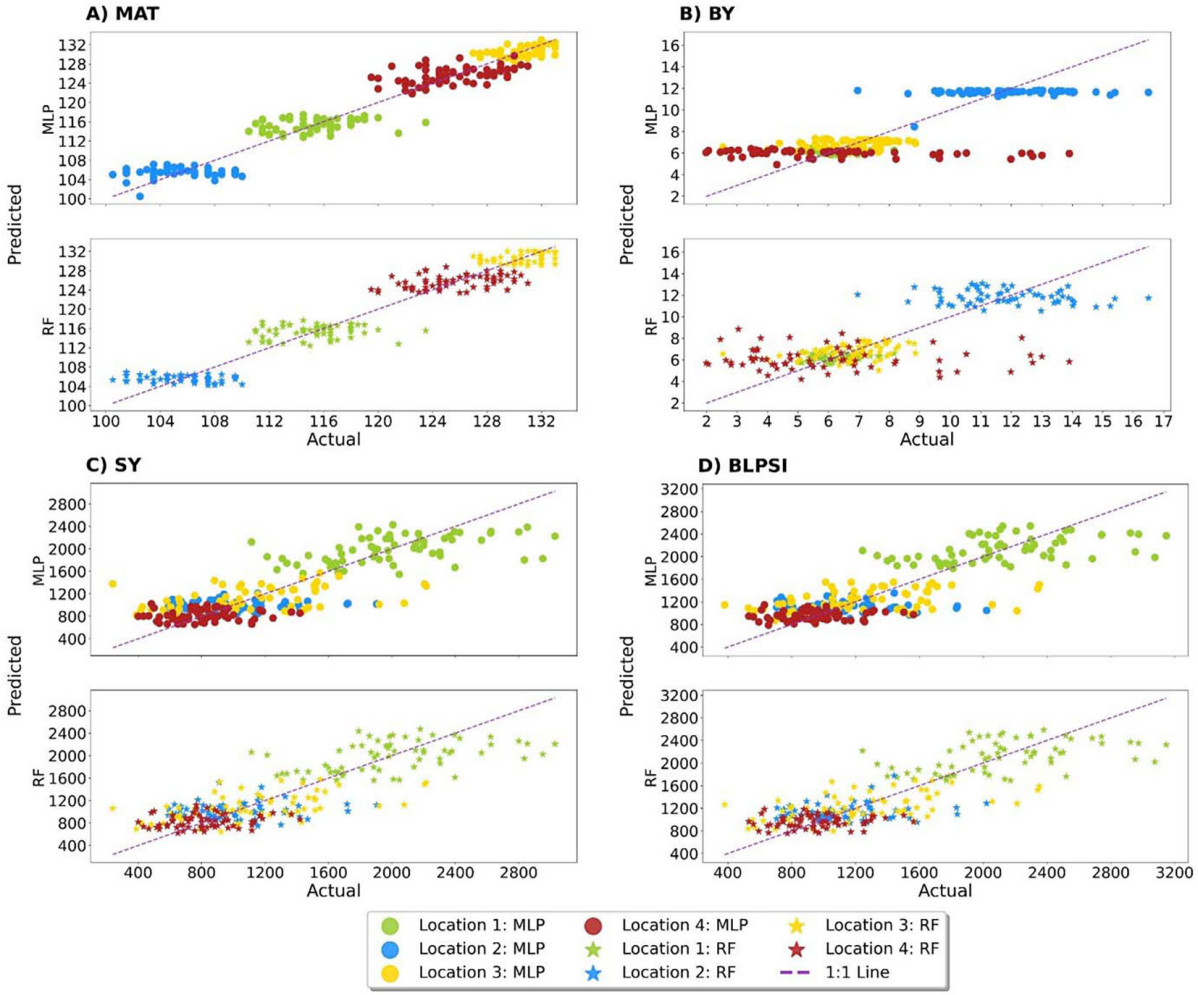
Actual versus predicted scores of grasspea genotypes obtained from the MLP and RF machine learning models across four traits. **(A)** Performance metrics of the utilized ML models for maturity (MAT), **(B)** Performance metrics of the utilized ML models for biological yield (BY), **(C)** Performance metrics of the utilized ML models for seed yield (SY), **(D)** Performance metrics of the utilized ML models for Base Linear Phenotypic Selection Index (BLPSI).

The present study revealed that the MLP model had the highest *R*^2^ scores, with values for maturity (MAT) and biological yield (BY) of 0.947 and 0.608, respectively. On the other hand, seed yield (SY) (0.682) and BLPSI (0.679) received the greatest *R*^2^ values from the RF model (**Table 5**; **Figure 9**). These scores indicate the extent to which the independent variables in the model can explain the variance in the dependent variable. Results for MAPE, RMSE, and MLSE were in line with the *R*^2^ scores, and the lowest scores for MAT and BY from the MLP model demonstrate its superior accuracy and predictive ability. With regard to SY and BLPSI, the RF model performed better as seen by its lower RMSE, MAPE, and MLSE scores. The performance of the MLP model was further validated using the MAE results. The MLP model demonstrated its accuracy in predicting MAT, BY, and SY by achieving the lowest MAE scores for these parameters. The MLSE results showed that both models’ responses to MAT were similar, indicating a similar performance for this metric. For BY, the MLSE score that the MLP model obtained was the lowest. For both SY and BLPSI, the RF model had the lowest MLSE score. The results of the MedAE showed how well the MLP model performed for all parameters.

**Table 5:** Performance metrics of the utilized ML models for different parameters.

MLP						
Traits*	*R* ^2^	RMSE	MAE	MAPE	MLSE	MedAE
MAT	0.947	2.304	1.797	1.539	0.000	1.515
BY	0.608	1.880	1.286	20.605	0.057	0.822
SY	0.674	328.307	245.249	24.646	0.091	187.791
BLPSI	0.661	333.412	254.337	21.304	0.069	198.004
RF						
Traits*	*R* ^2^	RMSE	MAE	MAPE	MLSE	MedAE
MAT	0.940	2.458	1.903	1.627	0.000	1.594
BY	0.558	1.995	1.392	22.131	0.063	0.895
SY	0.682	324.400	254.340	24.873	0.086	222.252
BLPSI	0.679	324.768	251.203	21.12	0.065	203.566

*MAT, days to maturity; BY, biological yield; SY, seed yield; BLPSI: Base Linear Phenotypic Selection Index.

For all parameters, the MLP model showed lower MedAE values than the RF model, indicating a higher overall predictive accuracy. Overall, the MLP model showed the lowest RMSE, MAPE, MLSE, MAE, and MedAE scores for these parameters, and the highest *R*^2^ scores for MAT and BY. In contrast, the RF model had the lowest RMSE, MAPE, MLSE, and MAE scores for SY and BLPSI along with the highest *R*^2^ scores. The MLP model demonstrated exceptional performance in MAE and MedAE, two crucial metrics for evaluating prediction accuracy and resilience.

## Discussion

Plant breeders frequently conduct multi-location trials (MLTs) to assess the test entries. This practice forms a strong foundation for the adoption and commercial release of varieties. Grasspea is a versatile crop concerning its wide adaptability (Das et al., 2021). The testing locations in the present study represent the diverse agro-climatic zones in India. In multi-locational trials, the variance components are mainly divided into three classes, main effect of genotype, environment, and their interaction (GE). The presence of the GE component adds ambiguity to genotype evaluation due to inconsistency in the performance of cultivars across different locations. Understanding the GEI is crucial for refining breeding strategies, selecting non-redundant testing sites, and recommending varieties for specific or broad adaptation. Over the past decade, Additive Main Effect and Multiplicative Interaction (AMMI) (Ahmadi et al., 2012; Rajendran et al., 2018; Rubiales et al., 2020) and the GGE biplot (Yan et al., 2007; Chatterjee et al., 2019) have gained prominence for the visual representation of GEIs, facilitating the identification of stable genotypes and optimal environments. The GGE biplot surpasses AMMI in ME analysis through elucidating a greater proportion of G+GE and providing insights into optimizing test environments through the “discrimination power vs. representativeness” graph (Yan et al., 2007). The present study attempted to cull out stable grasspea genotypes with a minimal GEI impact and a greater genetic buffering capacity employing the GGE biplot methodology.

In the present study, ANOVA confirmed the significant contributions of the environment and GEI for all the studied traits, which were considered for estimating the mean BLPSI, thereby corroborating the relevance of MLTs. Earlier literature (Gauch and Zobel, 1997) suggested that, in MLT data, the primary source of variation is the environment, accounting for over 80% of the total variation. In mungbean (Das et al., 2020; Singh et al., 2020), fieldpea (Das et al., 2019; Biswas et al., 2021), chickpea (Tamang et al., 2021), and lentil (Bhattacharya et al., 2022a, 2022b; Chatterjee et al., 2023), studies aligned with the current results, highlighting the prominence of environmental variation. In the current investigation, the substantial contribution of GEI was also evident, underscoring the presence of distinct MEs within the tested locations (Gore et al., 2021).

In MLTs, breeders must recommend genotypes based on combined yield-trait performance. However, most studies focus on single trait analysis, leaving other traits unexplored (Yan and Tinker, 2006). To address this, decisions must be taken considering the breeder’s experience and judgment for framing a selection index or subjective weight for multiple traits. In the present study, we have tried to visualize the GEI effect based on combined multi-trait performance of the grasspea genotypes wherein beside yield performance, maturity and biomass were also taken into consideration. It was observed that the performance of grasspea genotypes was unpredictable and inconsistent at different locations and ratified the presence of crossover interaction (COI). Genotypes G13, G33, G10, G63, and G6 showed promise in L1, while in L2, G32, G53, G21, G4, and G50 excelled. The presence of COI implied breeding for specific adaptation (Yan and Hunt, 2002; Rakshit et al., 2012). However, some genotypes (G13 and G33) exhibited non-COI, aligning with previous studies showing the presence of both COI and non-COI within the same datasets (Das et al., 2019; Singh et al., 2020). This complexity may diminish the genetic gain due to the intricacies during the selection procedure (Comstock and Moll, 1963).

The GGE biplot simplifies the complex interaction between the genotype, environment, and GEI in the form of different PCs, and their contribution justifies the usefulness of the methodology for explaining the sources of variation (Yan and Tinker, 2005). In this study, the two PCs explained more than 80% of the variation, which rationalized the adequacy of the MLT in grasspea. The ideal genotype selection in the GGE biplot is based on both mean performance and stability. In the present study, Prateek (G64) and 31-GP-F3-S7 (G33) were considered as the ideal genotypes due to their high BLPSI value considering multi-trait performance and minimal interaction with the environment in the form of less projection from “the AEC abscissa” (Yan and Falk, 2002). Genotypes that are close to the “ideal” genotypes are considered as “desirable” genotypes due to having their high genetic relationship with the “ideal” genotype (Yan and Tinker, 2005). 48-GP-F3-S3 (G14) was identified as a desirable genotype due to its close positional proximity with the ideal genotype. Genotype positioning along the AEC ordinate indicated performance variation across environments, likely influenced by weather parameters such as rainfall, temperature, and humidity (Dehghani et al., 2006). Thus, genotypes with low homeostasis exhibit high responsiveness towards environments.

Another important feature of the GGE biplot is the culling out of the optimal testing location judiciously with an aim of efficiently distributing resources, while also minimizing the overall trial costs. Importantly, this allocation strategy was designed to ensure that trial heritability and genetic gain under selection were not compromised in the process (Yan, 2001). Within the GGE framework, the projection of the vector onto the “AEC abscissa” plays a pivotal role in determining both the overall impact of the environment and the effectiveness of the methodology employed (Allen, 1978; Flores et al., 2013). In legume crops such as grasspea, where the additive component of variation holds a dominant role, the vector projection of the testing location on the GGE biplot serves as an indirect selection parameter, which is instrumental in confirming the effectiveness of the methodology being used (Yan and Holland, 2010). Taking all factors into account, L3 (Mohanpur) emerged as the optimal testing location due to its highest desirability index, being both discriminative and representative of the target environments. This selection makes it the ideal choice for evaluating advanced breeding materials, especially with a meager seed quantity that needs to be adjusted across various locations.

Additionally, the GGE biplot was able to separate all the testing locations into three MEs to aid the restructuring of agro-ecological zonation with the winning genotype for each center. An ME can be defined as a group of analogous locations delivering a similar genotypic response and sharing the same set of genotypes across the year (Yan and Rajcan, 2002). The “ideal” genotype identified in the present study, G13 (FLRP-B54-1-S2), was also the winning genotype in ME-I, while FLRP-B38-S5 (G10) for ME-II and IGC-2012-67/13-25 (G62) in ME-III revealed specific adaptation for the respective MEs. Prior research has utilized the GGE approach to assess testing locations and delineate specific environmental zones in a similar manner (Sayar and Han, 2015; Chatterjee et al., 2019; Vaezi et al., 2023).

The application of ML models in this study provided deeper insights into GEIs, improving predictive accuracy for key yield traits. The MLP model, with its ability to capture nonlinear relationships, performed well in predicting maturity and biological yield, benefiting from automated feature learning and multicollinearity handling (Sarker, 2021; Akay et al., 2022). Its strength in modeling continuous variables contributed to improved accuracy, particularly for traits influenced by multiple factors. Comparable integrative approaches using the GGE biplot and ML have been reported in cereals, providing useful benchmarks for our findings. For instance, Omrani et al. (2025) applied RF and MLP models alongside GGE analysis in wheat and found that combining multi-trait indices with predictive modeling improved genotype ranking stability compared to single-trait models, which is consistent with our observation that ML integration better captured genotype × environment patterns in grasspea.

Conversely, the RF model proved more effective in handling high-dimensional data, particularly for seed yield and soil-related traits (Wani et al., 2022). Its ensemble averaging approach reduced sensitivity to noise and outliers, resulting in more stable predictions. Additionally, its ability to rank feature importance provided valuable insights into the most influential agronomic factors (Fawagreh et al., 2014). The integration of ML with the GGE biplot approach further improved genotype selection for the Indian climate. ML models identified complex genotype × environment patterns, facilitating the selection of high-yielding and climate-adapted grasspea genotypes (Bello et al., 2015). This data-driven approach enhanced selection efficiency, making trait evaluation more precise and supporting climate-resilient breeding strategies.

The observed performance differences between MLP and RF highlight the importance of model selection based on trait characteristics. While MLP was effective for complex, continuous traits, RF provided robustness in high-dimensional datasets, making it particularly useful for trait selection and stability analysis. These findings emphasize the role of ML in improving prediction accuracy and decision-making in crop improvement programs. Thus, AI-driven tools can efficiently model and visualize these interactions, enabling researchers to identify genotypes with superior adaptability and yield potential in varied Indian climates. This approach makes data-driven decisions in genotype selection and breeding strategies, improving the efficiency and accuracy of identifying suitable grasspea genotypes.

## Conclusion

The present study highlighted the significant environmental and genotype × environment effects on grasspea performance across diverse Indian locations. GGE biplot analysis grouped the sites into three MEs, identifying FLRP-B54-1-S2, Prateek, and 31-GP-F3-S7 as ideal genotypes, and 48-GP-F3-S3 as a desirable genotype for targeted adaptation. ML models complemented traditional analyses, with MLP performing best for maturity and biomass, and RF for seed yield and BLPSI. This synergistic data-driven approach for genotype selection and identifying the ideal test environment can strengthen the substantial selection of location with optimization of resources in future breeding programs.

## Data Availability

The original contributions presented in the study are included in the article/**Supplementary Material**. Further inquiries can be directed to the corresponding authors.
