# Supplementary material for: Integrating machine learning and the GGE biplot for identification of climate-suitable grasspea genotypes
Source: Front Plant Sci. 2025 Nov 21;16:1647903. doi: 10.3389/fpls.2025.1647903 (PMC12679278; doi:10.3389/fpls.2025.1647903)
Supplement: Supplementary file 1 [file Table1.docx]

**Supplementary Table 1: Grasspea genotypes information**

| **Genotype Code** | **Genotype/**  **Environment** | **Status** | **Country of origin** |
| --- | --- | --- | --- |
| G1 | BANG-113-S5 | Germplasm | Bangladesh |
| G2 | 32-GP-F3-S2 | Germplasm | Nepal |
| G3 | BANG-147-S3 | Germplasm | Bangladesh |
| G4 | 32-GP-F3-S5 | Germplasm | Nepal |
| G5 | BANG-188-S4 | Germplasm | Bangladesh |
| G6 | 39-GP-F3-S2 | Germplasm | Nepal |
| G7 | BANG-277-S1 | Germplasm | Bangladesh |
| G8 | 40-GP-F3-S3 | Germplasm | Nepal |
| G9 | BANG-233-S1 | Germplasm | Bangladesh |
| G10 | FLRP-B38-S5 | Advanced breeding line | ICARDA |
| G11 | 40-GP-F3-S6 | Germplasm | Nepal |
| G12 | BANG-234-S1 | Germplasm | Bangladesh |
| G13 | FLRP-B54-1-S2 | Advanced breeding line | ICARDA |
| G14 | 48-GP-F3-S3 | Germplasm | Nepal |
| G15 | BANG-271-S2 | Germplasm | Bangladesh |
| G16 | 21-GP-F3-S5 | Germplasm | Nepal |
| G17 | 48-GP-F3-S10 | Germplasm | Nepal |
| G18 | BANG-288-S2 | Germplasm | Bangladesh |
| G19 | 23-GP-F3-S1 | Germplasm | Nepal |
| G20 | 48-GP-F3-S15 | Germplasm | Nepal |
| G21 | BANG-307-S2 | Germplasm | Bangladesh |
| G22 | 23-GP-F3-S2 | Germplasm | Nepal |
| G23 | 74-GP-F3-S1 | Germplasm | Nepal |
| G24 | BANG-307-S3 | Germplasm | Bangladesh |
| G25 | 23-GP-F3-S5 | Germplasm | Nepal |
| G26 | 74-GP-F3-S5 | Germplasm | Nepal |
| G27 | BANG-27-S2 | Germplasm | Bangladesh |
| G28 | 25-GP-F3-S3 | Germplasm | Nepal |
| G29 | BANG-31-S6 | Germplasm | Bangladesh |
| G30 | 31-GP-F3-S2 | Germplasm | Nepal |
| G31 | BANG-15-S1 | Germplasm | Bangladesh |
| G32 | 31-GP-F3-S4 | Germplasm | Nepal |
| G33 | 31-GP-F3-S7 | Germplasm | Nepal |
| G34 | IGC-2012-70/1-8 | Advanced breeding line | ICARDA |
| G35 | IGC-2012-31/2-37 | Advanced breeding line | ICARDA |
| G36 | IGC-2012-6/3-36 | Advanced breeding line | ICARDA |
| G37 | IGC-2012-78/4-19 | Advanced breeding line | ICARDA |
| G38 | IGC-2012-76/5-42 | Advanced breeding line | ICARDA |
| G39 | IGC-2012-4/6-50 | Advanced breeding line | ICARDA |
| G40 | IGC-2012-2/8-8 | Advanced breeding line | ICARDA |
| G41 | IGC-2012-74/10-7 | Advanced breeding line | ICARDA |
| G42 | IGC-2012-88/11-50 | Advanced breeding line | ICARDA |
| G43 | IGC-2012-24/12-43 | Advanced breeding line | ICARDA |
| G44 | IGC-2012-31/2-1 | Advanced breeding line | ICARDA |
| G45 | IGC-2012-6/3-42 | Advanced breeding line | ICARDA |
| G46 | IGC-2012-78/4-5 | Advanced breeding line | ICARDA |
| G47 | IGC-2012-76/5-14 | Advanced breeding line | ICARDA |
| G48 | IGC-2012-4/6-8 | Advanced breeding line | ICARDA |
| G49 | IGC-2012-74/10-41 | Advanced breeding line | ICARDA |
| G50 | IGC-2012-24/12-26 | Advanced breeding line | ICARDA |
| G51 | IGC-2012-70/1-5 | Advanced breeding line | ICARDA |
| G52 | IGC-2012-6/3-39 | Advanced breeding line | ICARDA |
| G53 | IGC-2012-31/2-44 | Advanced breeding line | ICARDA |
| G54 | IGC-2012-6/3-47 | Advanced breeding line | ICARDA |
| G55 | IGC-2012-4/6-23 | Advanced breeding line | ICARDA |
| G56 | IGC-2012-14/7-44 | Advanced breeding line | ICARDA |
| G57 | IGC-2012-2/8-35 | Advanced breeding line | ICARDA |
| G58 | IGC-2012-73/9-5 | Advanced breeding line | ICARDA |
| G59 | IGC-2012-74/10-1 | Advanced breeding line | ICARDA |
| G60 | IGC-2012-6/3-43 | Advanced breeding line | ICARDA |
| G61 | IGC-2012-24/12-24 | Advanced breeding line | ICARDA |
| G62 | IGC-2012-67/13-25 | Advanced breeding line | ICARDA |
| G63 | Mahateora | Released variety | India |
| G64 | Prateek | Released variety | India |
